# A fatal case of acute progression of generalized edema and simultaneous flash pulmonary edema in a patient with idiopathic systemic capillary leak syndrome: a case report

**DOI:** 10.1186/s13256-015-0544-5

**Published:** 2015-04-28

**Authors:** Yuri Hirosaki, Shunji Hayashidani, Sayako Ouchi, Tukasa Ohshima, Ryuji Nakano, Hideo Yamamoto

**Affiliations:** 1 Department of Internal Medicine, Japan Community Health Care Organization (JCHO) Kyushu Hospital, 1-8-1 Kishinoura Yahata-Nishiku, Kitakyushu City, Fukuoka Prefecture 806-8501 Japan; 2 Department of Pathology, Japan Community Health Care Organization (JCHO) Kyushu Hospital, 1-8-1 Kishinoura Yahata-Nishiku, Kitakyushu City, Fukuoka Prefecture 806-8501 Japan

**Keywords:** Systemic capillary leak syndrome, Hypovolemic shock, Pulmonary edema, Autopsy findings

## Abstract

**Introduction:**

Idiopathic systemic capillary leak syndrome is a rare and fatal disease due to the unexplained episodic attacks of capillary leakage of plasma from the intravascular into the interstitial space. The attack consists of three phases, a prodromal phase, peripheral leak phase and recruitment phase. During the peripheral leak phase, generalized edema, mainly in the trunk and extremities, with hemoconcentration and hypoalbuminemia occurs, while usually the visceral organs like lungs, brain, heart and kidneys seem not to be involved. Treatment of the acute phase is supportive, focusing on adequate but not overzealous fluid resuscitation, because pulmonary edema usually occurs in the recruitment phase.

**Case presentation:**

A 65-year-old Japanese woman was admitted to our hospital because of severe hypovolemic shock with metabolic acidosis and hemoconcentration and hypoalbuminemia. Although she was considered to be in the peripheral leak phase of idiopathic systemic capillary leak syndrome, which could not be diagnosed during the treatment, the generalized edema worsened further, severe flash pulmonary edema progressed rapidly after fluid resuscitation and she died. The autopsy showed generalized edema, especially alveolar pulmonary edema without endothelial apoptosis.

**Conclusions:**

Because hypovolemic shock and fatal pulmonary edema may progress rapidly together even in the peripheral leak phase of idiopathic systemic capillary leak syndrome, we should keep in mind this rare and fatal disease and recognize the pathophysiology to treat it effectively when the patient has hypovolemia with metabolic acidosis.

## Introduction

Idiopathic systemic capillary leak syndrome (SCLS) is a very rare disease with a high mortality rate. SCLS was first described by Clarkson in 1960 [1]. Since then about 160 cases have been described [2-5]. The syndrome is characterized by unexplained increased capillary permeability resulting in generalized edema and hypovolemia with the SCLS trio of hypotension, hemoconcentration and hypoalbuminemia due to the marked shift of intravascular fluid (up to 70%) and macromolecules like albumin from the intravascular to the extravascular space [3]. The diagnosis of SCLS is performed clinically by the exclusion of other diseases that cause systemic capillary leakage. The mortality rate among SCLS patients has been reported to be as high as 18 to 36% [5-8]. In one report, 75% of the deaths were directly related to SCLS attacks [5]. We describe a hypovolemic shock patient who was hospitalized without a definitive diagnosis and died from the first severe attack of SCLS. SCLS is a very rare and fatal disease, and so it is very important to frequently emphasize the necessity of recognizing the pathophysiology of SCLS [9].

## Case presentation

**Figure 1:**
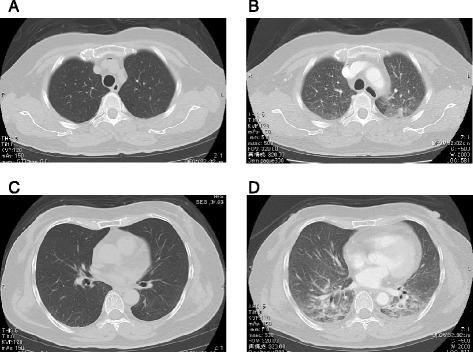
Computed tomography scans. **(A, C)** The plain computed tomography scan on admission. **(B, D)** The contrast computed tomography scan at hemodynamic deterioration. A and B were the same level of the body. C and D were the same level of the body.

**Figure 2:**
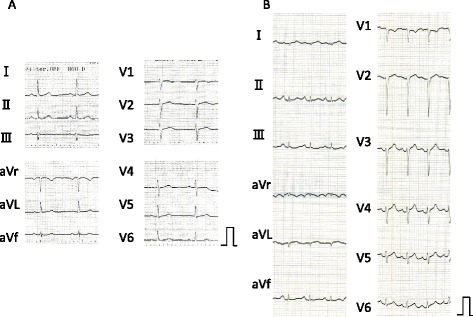
Electrocardiograms. **(A)** Electrocardiogram, one month before admission. **(B)** Electrocardiogram on admission.

**Figure 3:**
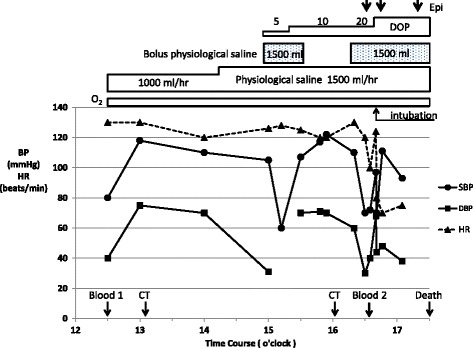
Clinical course and treatment. Black circle, systolic blood pressure (mmHg); black square, diastolic blood pressure (mmHg); black triangle, heart rate (beats/min); Blood 1, first collecting blood for laboratory tests; Blood 2, second collecting blood for laboratory tests; BP, blood pressure; CT, computed tomography; DOP, dopamine (mcg/kg/min); Epi, 1mg bolus intravenous administration of epinephrine; HR, heart rate; O_2_, oxygen supply (3 to 8L/min).

A 65-year-old Japanese woman was admitted to our hospital because of face and limb edema, back pain and severe general fatigue. She had complained of the symptoms of a cold with fever one week before admission. Her consciousness was mildly disturbed, her blood pressure was 80/40mmHg, heart rate was 130/min, respiratory rate was 32 breaths/min and her body temperature was 35.6°C. Lymphadenopathy was not detected. Skin flush, urticaria, focal angioedema and stridor were not observed. Her breath sounds and heart sounds were normal. An abdominal examination showed cyanotic skin and slight tenderness on the right lower quadrant. Her extremities were cold and markedly edematous. Laboratory data obtained on admission showed the following values: white blood cells 30,600/μL with a normal fraction, red blood cells 688×10^4^/μL, hematocrit 67.2%, hemoglobin 22.1g/dL, C-reactive protein 4.6mg/dL, blood urea nitrogen 43mg/dL, creatinine 2.2mg/dL, total protein 5.5g/dL, albumin 3.1g/dL, aspartate aminotransferase (AST) 34IU/L, alanine transaminase (ALT) 25IU/L, alkaline phosphatase (ALP) 220IU/L, lactate dehydrogenase (LDH) 363IU/L, creatine phosphokinase (CPK) 243IU/L, creatine kinase MB (CK-MB) 27.0IU/L, D-dimer 0.8μg/mL, blood glucose 428mg/dL, sodium 130mEq/L, potassium 5.1mEq/L, and HbA1c 6.2%. The laboratory test performed later showed that monoclonal immunoglobulin G (IgG), rheumatoid factor, antinuclear antibody, and vascular endothelial growth factor (VEGF) (<20pg/ml) were undetectable. Her arterial blood gas analysis showed metabolic acidosis (pH: 6.95, PO_2_: 102mmHg, PCO_2_: 21.3mmHg, HCO_3_^−^: 4.4mmol/L, BE: −35.9mmol/L). A chest X-ray film was normal and a plain computed tomography (CT) scan revealed no pleural effusion, pulmonary congestion nor cardiomegaly but mild pericardial effusion (Figure 1A, C). An electrocardiogram (ECG, Figure 2B) showed the low voltage of the limb leads, QS pattern in I and aVL, poor R wave progression in V1 to 4 and ST elevation in V4 to 6, which were not found in the electrocardiogram recorded one month previously (Figure 2A). A portable echocardiography examination was normal except for mild pericardial effusion and the decreased diameter of the inferior vena cava. At this time, we thought the severe hemoconcentration indicated intravascular hypovolemia, which had caused the peripheral circulatory disturbances and the metabolic acidosis. We started infusing extracellular fluids from a central vein with intravenous administration of insulin according to the sliding scale to correct the intravascular volume deficit and to restore hemodynamic stability (Figure 3).

In spite of continuous intravenous infusion (total about 2000mL), her blood pressure dropped gradually and about two hours later dropped suddenly to be undetectable, but she could respond to our call. The more rapid infusion of the extracellular fluid (about 1500mL) from a central vein with use of the catecholamine (dopamine 5 to 10mcg/kg/min) restored her blood pressure 30 minutes later and then the contrast CT scan was taken. It did not show any findings of pulmonary embolism and acute abdomen such as acute pancreatitis, abscess, and mesentelic arterial occlusion, but it showed pulmonary congestion and massive edema of the whole body (Figure 1B, D), which were not found by the first plain CT scan. At this time, the portable echocardiography showed normal left ventricular wall motion. One hour after that, hemodynamic deterioration and respiratory failure due to severe pulmonary edema progressed rapidly. Intratracheal intubation was done but the water blew out from the tube and she died about one hour later in spite of the aggressive treatment (Figure 3). The laboratory test result on this hemodynamic deterioration was reported after her death and showed the significant progression of the hypoalbuminemia on the following laboratory data: white blood cells 38,000/μL with a normal fraction, red blood cells 507×10^4^/μL, hematocrit 45.6%, hemoglobin 15.7g/dL, blood urea nitrogen 38mg/dL, creatinine 2.3mg/dL, total protein 1.2g/dL, albumin 0.6g/dL, AST 49IU/L, ALT 35IU/L, ALP 100IU/L, LDH 328IU/L, CPK 543IU/L, blood glucose 226mg/dL, sodium 154mEq/L, potassium 3.9mEq/L. Blood cultures were negative.

The autopsy showed generalized edema, especially alveolar pulmonary edema without endothelial apoptosis. The pulmonary alveolar spaces were filled with fluids, which were not stained so much by hematoxylin and eosin. The coronary arteries did not have any atherosclerotic stenosis. Inflammatory cells were not found in the edematous tissues. The bone marrow showed no evidence of multiple myeloma. There were no signs of infection.

She had a medical history of two previous episodes of generalized edema and fatigue after antecedent flu-like illness. She recovered without any medication. One month before admission, the laboratory data of her regular medical check was normal as per the following values: hemoglobin 12.7g/dL, protein 6.9g/dL, creatinine 0.78mg/dL, and blood urea nitrogen 14.8mg/dL. After her death, we diagnosed that our patient had had SCLS in consideration of all these medical histories.

## Discussion

We describe a 65-year-old woman who was hospitalized because of severe hypovolemic shock with hemoconcentration and hypoalbuminemia. Our case was similar to other reports describing patients with SCLS and was diagnosed as severe SCLS according to the report by Gousseff *et al.* [5]. SCLS is diagnosed clinically after exclusion of other diseases that cause systemic capillary leakage, including severe sepsis, toxic shock syndrome and anaphylaxis. In our patient, blood cultures were negative and there were no findings of infection by autopsy, which indicated she had not had sepsis. Her cutaneous and respiratory findings, and laboratory examinations, showed that she had not had toxic shock syndrome and anaphylaxis.

SCLS attack consists of three phases, a prodromal phase, peripheral leak phase and recruitment phase [3]. About 30% of SCLS patients show an antecedent upper respiratory tract infection or a flu-like illness with fever in the prodromal phase, like our patient. One to four days after the prodromal phase, the capillary leakage develops and results in generalized edema, mainly in the trunk and extremities, while the visceral organs like lungs, brain, heart and kidneys usually seem not to be involved [3,5]. Some patients have hypovolemic shock and ischemic end-organ damage, which include acute kidney injury, ischemic brain injury and ischemic hepatic dysfunction. The peripheral leak phase continues for several days and the recruitment phase begins quickly, in which massive fluid and macromolecules are recruited back from the third space into the circulation. The patient is at high risk for intravascular volume overload and pulmonary edema during this recruitment phase. The reported cases of the acute phase of SCLS in the literature (from 1990 to 2011, [2,7,8,10-16]) are summarized in Table 1. Five out of 13 patients had pulmonary edema, which occurred in the recruitment phase in four of them and the other one patient had it in the peripheral leak phase because of the iatrogenic effect of the large amount of transfusion (case 10). Kapoor *et al.* evaluated 25 patients with SCLS to determine clinical features, natural history and outcome [4] Pulmonary edema developed in 10 out of 25 patients (40%) during the recovery phase in the face of ongoing vigorous fluid resuscitation. Therefore, in the peripheral leak phase, adequate organ perfusion has to be obtained by sequential rapid and large infusion of intravascular fluids if hypoperfusion exists. In the recruitment phase, control of the fluid overload must be performed with the use of diuretics and sometimes by mechanical ventilation [3].

**Table 1:** **Reported cases of the acute phase of idiopathic systemic capillary leak syndrome**

**Case (reference)**	**Age (yrs)**	**Sex**	**SBP (mmHg)**	**HR (beats/min)**	**TP / Alb (g/dL)**	**Ht / Hb (g/dL)**	**Pulmonary edema**	**Intubation**	**Treatment**	**Outcome**
1 [2]	46	M	56	96	4.3/2.5	61.7% / 21.9	+ (day 2, R.P.)	+(day 3)	Saline: 10L/12hr, Alb	A
2 [7]	49	F	Shock	Shock	None	None	-	-	Saline	A
3 [7]	54	M	Shock	Shock	3.7 / None	56% / None	-	-	Saline: 4.5L+Alb	A
4 [7]	44	F	Shock	Shock	None	None	-	-	None	A
5 [8]	24	M	80	130	5.5 / 3.0	None / 12	-	-	Saline: 1L+0.5L/hr+C	A
6 [10]	52	F	Shock	Shock	None	67% / None	+(R.P.)	-	Plasma expander: 1500mL/24hrs	A
7 [11]	53	M	Shock	100	None / 1.3	67% / None	-	-	Saline: 2L+3L/48hr	A
8 [12]	38	F	80	None	4.4 / None	49.9% / 16.8	-	-	Saline: 4.5L+Alb+C	A
9 [13]	37	F	40	110	None	53% / 19.2	+(day 1)	+	Saline:9L+10% P+S0.5L/hr+C	A
10 [13]	36	F	40	130	4.4 / None	49.9% / 16.8	-	-	10% P+S	A
11 [14]	41	M	70	40	None / 1.8	None / 22.5	+(day 3, R.P.)	+	Saline: 15L, Alb+C	A
12 [15]	41	M	65	102	2.3 / none	60.4% / 22.3	-	+	Saline: 15L+Nad	A
13 [16]	62	F	97	None	None	None / 19.7	+ (day 5)	+ (day 5)	Saline+C+IMG+S	D (day 9)

SBP, systolic blood pressure; HR, heart rate; TP, total protein; Alb, albumin; Ht, hematocrit; Hb, hemoglobin; F, female; M, male; None, data not described; R.P., recruitment phase; C, catecholamine; P, pentastarch; S, steroid; Nad, noradrenaline; IMG, immunoglobulins; A, alive; D, death.

In SCLS, the endothelial barrier dysfunction leads to leakage of the intravascular fluid and macromolecules like albumin into the interstitial space. Although crystalloids are used commonly as intravascular fluids, colloid (albumin) also should be used for the intravascular fluid resuscitation in SCLS, because colloid increases intravascular oncotic pressure and keeps the fluid in the intravascular space for a longer period than crystalloid alone [3]. However, proteins like albumin, with a molecular weight less than 200 kilodaltons, leak from the vessels in SCLS and the effectiveness of colloid may not be enough. Recently, it was reported that the infusion of 10% pentastarch, a larger molecule than albumin, had the effect to stabilize the hemodynamics in two SCLS patients with refractory hypotension during the peripheral leak phase, who did not respond to aggressive crystalloid replacement and inotropic agents [13]. The pharmacologic therapies to improve the capillary leakage were tried in the acute phase of SCLS. Terbutaline, by inducing adenyl cyclase, and theophylline, by inhibiting phosphodiesterase, increase the cyclic adenosine monophosphate (cAMP) levels of the endothelium. The increase in cAMP is known to decrease endothelial cell permeability [17], and so both are supposed to interfere with capillary permeability. Beta-2 stimulators like terbutaline can also inhibit macromolecular leakage in response to histamine and bradikynin [18]. Theophylline also is known to antagonize cytokine-mediated endothelial damage and capillary permeability [19]. Through these pharmacologic activities, both terbutaline and theophylline are used as a first-line prophylactic therapy against chronic SCLS [20]. In three patients with acute phase SCLS, Dowden *et al.* showed that the combination of terbutaline and theophylline had beneficial effects by decreasing capillary permeability if the serum theophylline level was high enough (20 to 25mcg/mL), and that if it did not work, the additional administration of tumor necrosis factor alpha (TNF-alpha) antagonist (infliximab) seemed to improve the patient refractory to terbutaline and theophylline [21]. Lambert *et al.* reported high-dose intravenous immunoglobulins were effective against the acute phase of SCLS [22]. VEGF was reported in 1983 to induce rapid leakage from blood vessels [23]. Recently, VEGF was reported to increase in the episodic SCLS active phase but not in the remission phase [24,25]. Yabe *et al.* reported that anti-VEGF antibody (bevacizumab) improved the life-threatening acute phase of SCLC [26]. However, we need to be aware of SCLS and to diagnose it before using them, because these acute pharmacologic challenges are not established and a TNF-alpha antagonist is dangerous if patients have sepsis.

Our patient was hospitalized in the peripheral leak phase of SCLS with prolonged hypoperfusion, because she had distributive shock with metabolic acidosis. Although we did not have a definitive diagnosis, we started infusing extracellular fluid (crystalloid) without colloid, and her hemodynamics improved transiently. Usually, the lungs may not be involved in the peripheral leak phase [3]. But during the extracellular fluid infusion, the generalized edema progressed and the severe flash pulmonary edema also progressed rapidly, and she died (Figure 3). There might be several possible explanations. The rapid decrease in oncotic pressure due to severely progressing hypoalbuminemia and the increase in the pulmonary artery resistance due to metabolic acidosis [27,28] and the aggressive extravascular fluid resuscitation without colloid, all of them might induce flash pulmonary edema. Although we thought the serum albumin level of 3.1g/dL on admission was mild, its level had to have been actually very low in consideration of her severe hemoconcentration. The degree of hypovolemia and systemic tissue hypoperfusion on admission were also severe in consideration of her metabolic acidosis and hemoconcentration, and so the pulmonary capillary hydrostatic pressure was low enough not to induce pulmonary edema in spite of her hypoalbuminemia on admission. Therefore, we should have understood the pathogenesis of the SCLS and have transfused the appropriate dose of colloids with crystalloids while monitoring the central venous pressure on admission and performed mechanical ventilation with positive end-expiratory pressure (PEEP) at the latest at the occurrence of the first hemodynamic deterioration. The myocardial involvement associated with the SCLS might be another possibility [11,29,30]. Claessens *et al.* showed the reversible biventricular wall thickening, while systolic function remained normal by echocardiography. The ECG revealed cardiac axial rotation of −30° with nonspecific repolarization abnormalities in the attacks of SCLS, as in our patient [11]. Juthier *et al.* observed both myocardial thickening and severe systolic dysfunction by echocardiography, and diffuse myocardial interstitial edema without inflammatory infiltrates and myocyte necrosis by endomyocardial biopsies in the shock stage in one patient with SCLS [29]. In our patient, the autopsy findings did not show myocardial interstitial edema.

The pathogenesis of SCLS is unknown. Some hypotheses have been proposed, but hypotheses have incomplete evidence [3]. About 80% of patients with SCLS have a monoclonal immunoglobulin in their serum and these paraproteins might be related to the endothelial damage and vascular leakage [5,8,31]. But our patient did not have a monoclonal immunoglobulin. Some reports proposed the role of the cytokines, because interleukin 2 (IL-2)-receptor positive cells and cluster of differentiation 8 (CD8)-positive lymphocytes were found surrounding damaged endothelial cells during attacks in one patient [32]. However, in our patient, we could not find the lymphocytes around the capillaries of the autopsy tissues. VEGF is considered to be related to the pathogenesis of SCLS [23-25]. In our patient, VEGF was within normal limit. Therefore, the etiology of our patient remains unknown.

## Conclusions

It is important to keep in mind this rare and fatal disease of SCLS and recognize the pathophysiology to treat it effectively because severe hypovolemic shock and severe flash pulmonary edema may progress rapidly together during fluid resuscitation even in the peripheral leak phase of SCLS. To prevent these situations, intubation and mechanical ventilation with PEEP and fluid resuscitation by appropriate dose of crystalloids with colloids should be done while monitoring the central venous pressure when hypoalbuminemia and systemic tissue hypoperfusion are considered to be severe from the grade of hemoconcentration and metabolic acidosis.

## Consent

Written informed consent was obtained from the patient’s family for publication of this case report and any accompanying images. A copy of the written consent is available for review by the Editor-in-Chief of this journal.

## Abbreviations

ALP: alkaline phosphatase
ALT: alanine transaminase
AST: aspartate aminotransferase
cAMP: cyclic adenosine monophosphate
CD8: cluster of differentiation 8
CK-MB: creatine kinase MB
CPK: creatine phosphokinase
CT: computed tomography
ECG: electrocardiogram
IgG: immunoglobulin G
IL-2: interleukin 2
LDH: lactate dehydrogenase
PEEP: positive end-expiratory pressure
SCLS: idiopathic systemic capillary leak syndrome
TNF-alpha: tumor necrosis factor alpha
VEGF: vascular endothelial growth factor
